# A cross-sectional study on demoralization in prostate cancer patients: the role of masculine self-esteem, depression, and resilience

**DOI:** 10.1007/s00520-022-07145-9

**Published:** 2022-05-18

**Authors:** Cristiano Scandurra, Francesco Mangiapia, Roberto La Rocca, Francesco Di Bello, Natascia De Lucia, Benedetta Muzii, Micaela Cantone, Rita Zampi, Gianluigi Califano, Nelson Mauro Maldonato, Nicola Longo

**Affiliations:** 1 Department of Neuroscience, Reproductive Sciences and Dentistry, University of Naples Federico II, Via Sergio Pansini 5, 80131 Naples, Italy; 2 Department of Humanistic Studies, University of Naples Federico II, Via Porta di Massa 1, 80133 Naples, Italy; 3 Departmental Program of Clinical Psychopatology, Public Hospital of Naples Federico II, Via Sergio Pansini 5, 80131 Naples, Italy

**Keywords:** Prostate cancer, Demoralization, Depression, Masculine self-esteem, Resilience

## Abstract

**Purpose:**

The current cross-sectional study had three objectives: (1) to assess the prevalence of depression and demoralization in a sample of prostate cancer (PCa) patients; (2) to examine whether masculine self-esteem and depression were associated with demoralization; and (3) to evaluate the role of resilience as a factor buffering the effects of masculine self-esteem and depression on demoralization.

**Methods:**

197 PCa patients aged 48 to 79 years (*M* = 67.19; *SD* = 6.83) answered questions about masculine self-esteem, depression, resilience, and demoralization. An ANOVA was conducted to examine whether the association between demoralization and depressive symptoms was linear. A chi-square test was calculated to determine differences between depression and demoralization. Finally, a hierarchical multiple linear regression analysis with interaction terms was conducted to examine the associations between masculine self-esteem, depression, resilience, and demoralization.

**Results:**

Depression scores increased linearly with demoralization severity, but demoralization scores were higher than depression scores (21.3% vs. 15.2%). Lower scores on masculine self-esteem and higher scores on depressive symptoms were associated with greater demoralization. Resilience significantly moderated the association between masculine self-esteem and demoralization, but not between depression and demoralization.

**Conclusion:**

Assessment of depression, masculine self-esteem, resilience, and demoralization in the clinical setting is critical for improving the mental health status of PCa patients.

## Introduction

Prostate cancer (PCa) is the second most common solid cancer and the fifth leading cause of cancer death in men worldwide [1]. The effects of radical treatment of PCa on both physical (e.g., urinary, bowel, and sexual dysfunction) [2] and mental health (e.g., depression, anxiety, and suicidal ideation) [3] are well known.

Predictive risk factors for negative mental health outcomes have been studied mainly with respect to depressive symptoms. Fervaha et al. [4] reported that risk factors for depression in PCa patients can be classified as both biological (e.g., advanced stage, greater burden of physical symptoms, and older age) and psycho-social (e.g., helplessness, low family support, not being partnered, and a personal history of psychiatric illness). Similarly, Erim et al. [5] found that the most predictive factors for depression were ethnicity (i.e., not being Caucasian), unemployment, low annual income, past depression, comorbidities, treatment decisional regret, and nonadherence to exercise recommendations. Regarding suicidal ideation, Recklitis et al. [6] found that it was significantly associated with employment status, poor physical and emotional functioning, greater symptom burden, higher frequency of significant pain, and depression.

A specific risk factor for negative mental health outcomes in the PCa population has been identified in masculine self-esteem [7], or rather, in the way men appraise their masculinity after treatment for PCa. Indeed, such treatments generally lead to changes in urinary continence, sexual functioning, and energy levels [4, 8, 9], and this may affect self-perception as a “whole man” [10]. In this context, previous studies reported that one-third of men tend to have low masculine self‐esteem after treatment [11, 12], which in turn is associated with higher anxiety and depression and lower quality of life [7, 10].

Beyond risk factors, cancer patients are able to protect themselves from the negative mental health outcomes by activating psychological resources. Resilience, the ability to adapt to and recover from adversity, plays a crucial role in this process [13], being a powerful protective factor against depression and poorer quality of life in PCa patients [14], but also in patients with other types of cancer [15, 16].

However, in the last two decades, psycho-oncological research has highlighted the crucial difference between depression and demoralization [17]. Specifically, the core symptoms of depression include a loss of pleasure and interest in the present (i.e., anhedonia), whereas the symptoms of demoralization are a loss of hope and meaning, with a loss of anticipatory pleasure rather than a general anhedonia (i.e., helplessness, hopelessness, and meaninglessness) [18, 19]. This means that a substantial number of medically ill patients do not develop clinical depression, but rather a desire to die based on suicidal ideation [17]. Robinson et al. [17] reported that the prevalence of demoralization syndrome in patients with progressive disease and cancer patients ranges from 13 to 18%, and that poorly controlled physical symptoms, inadequately treated depression and anxiety, decreased social functioning, unemployment, and single status are predictive factors. Both demoralization and depression are common in cancer [20] and have been associated with suicidal ideation [21]. However, Nanni et al. [22] found that, among cancer patients who had suicidal ideation, 25% were demoralized but not clinically depressed, representing a crucial difference between the two mental states. In this context, recent studies have shown that demoralization has a greater impact on suicidal ideation than depression [23, 24]. Within this promising framework, Liu et al. [25] found that hopelessness influences suicidal ideation through both direct and indirect effects, or rather through the effect of demoralization or demoralization along with depression, but not only through depression. Although recent research has addressed the demoralization syndrome in cancer patients, this syndrome is not well known among oncology physicians, who seem to tend to confuse demoralization with depression [26].

To our knowledge, no previous studies have investigated demoralization in PCa patients. Therefore, the current study had three main objectives: (1) to assess the prevalence of depression and demoralization in a group of PCa patients; (2) to examine whether masculine self-esteem and depression were associated with demoralization; and (3) to evaluate the role of resilience as a factor buffering the effects of masculine self-esteem and depression on demoralization. We hypothesized that: (1) rates of demoralization would be higher than rates of depressive symptoms; (2) masculine self-esteem problems and depressive symptoms would be positively associated with demoralization; and (3) resilience would moderate the relationship of problems in masculine self-esteem and depressive symptoms with demoralization, by reducing the effects of these two dimensions on demoralization.

## Methods

### Procedures

This was a cross-sectional study conducted in the Urological Department of the University Hospital of Naples Federico II. All PCa patients who had visited the department between 2013 and 2021 were contacted by email and asked to participate in the study by completing an online survey uploaded on Google. All patients admitted to the department received a consent form requesting some personal information (e.g., email) and asking permission to be contacted for research purposes. Thus, a list of patients was generated through the department’s database. The email informed potential participants of the survey and that it would be available from January to March 2022.

In addition, participants were asked to provide their first and last names so that researchers could complete a clinician-report to match the questionnaires with some medical information. To this end, participants were informed of the following procedures: (1) data were protected by a secure gateway to which only the principal investigator (PI; i.e., the first author of the current study) had access; (2) the PI stored participants’ names and surnames on a separate sheet; (3) the PI shared this sheet with a urologist who provided the medical information; (4) the urologist shared this sheet with the PI, who created a unique dataset containing all the information; and (5) finally, the PI deleted all participants’ personal information before sharing the dataset with other researchers.

To avoid problems of social desirability, we emphasized in more than one part of the survey that PI did not know any patients and that he had an ethical obligation not to share private information with other investigators and especially with urologists who might have recognized patients.

By clicking on the link provided, participants were directed to the consent form and informed of the study objectives, benefits, and risks. To avoid missing data, all questions had to be answered, but participants were informed of their right to stop the survey at any time and for any reason. To participate in the study, patients were required to give their consent to participate by clicking “I accept to participate in the study.” Only after the participants agreed to participate could they answer the questionnaires, which were all in Italian.

The study was approved by the ethical committee of the University of Naples Federico II (protocol number 261/2019) and designed in accordance with the Declaration of Helsinki on Ethical Principles for Medical Research Involving Human Subjects.

### Participants

Participants were considered eligible if they (1) were over 18 years of age; (2) had a histologically confirmed PCa diagnosis; (3) had undergone radical prostatectomy or radiotherapy for clinically localized PCa; and (4) were able to understand and sign the informed consent form and complete the questionnaire independently.

Based on the above inclusion criteria, 286 patients were contacted by email and 197 participated in the study (response rate: 68.8%).

The age of the patients ranged from 48 to 79 years (*M* = 67.19; *SD* = 6.83). Most patients were Caucasian (*n* = 193; 98%), heterosexual (*n* = 191; 96.9%), with an education level ≤ high school (*n* = 148; 75.1%), and in a stable relationship (*n* = 167; 84.8%).

### Measures

#### Sociodemographic and clinical characteristics

The following variables of PCa patients included in the study were recorded: age, ethnicity (Caucasian vs. non-Caucasian), education level (≤ high school vs. ≥ university), marital status (with partner vs. without partner), timing of intervention (from 2013 to 2021), primary treatment (surgery only vs. radiotherapy only vs. surgery in combination with androgen deprivation therapy [ADT] vs. radiotherapy in combination with surgery), ISUP (International Society of Urological Pathology) grade (from 1 to 5), serum level of prostate-specific antigen (PSA) (ng/ml), and Gleason score of the radical prostatectomy specimen reported with the five-tiered Gleason Grade Groups (GGG) [27].

#### Depressive symptoms

The *Patient Health Questionnaire Depression Scale-9* (PHQ-9) [28] was used to measure the severity of participants’ depressive symptoms. The PHQ-9 consists of 9 items that ask about the frequency of depressive symptoms experienced in the past 2 weeks, with higher scores reflecting more severe depressive symptomatology. Each item is scored on a 4-point Likert scale, ranging from 0 (not at all) to 2 (nearly every day). A cut-off score of ≥ 10 is generally used as a screening method for major depressive disorder [29]. Recently, Ignatius et al. [30] classified the PHQ-9 into 3 domains: low depression (scores 0 ~ 9), moderate depression (scores 10 ~ 14), and high depression (scores 15 ~ 27). The *α* coefficient for the current sample was 0.84.

#### Masculine Self-Esteem Scale

The *Masculine Self-Esteem Scale* (MSES) [31] is a measure used specifically for PCa patients to assess the physical and mental components associated with negative self-appraisal related to masculinity. It consists of 8 items on a 5-point Likert scale ranging from 1 (none) to 5 (very much). Higher scores indicate greater self-esteem. The *α* coefficient for the current sample was 0.91.

#### Resilience

Resilience was measured using the *Resilience Scale* (RS) [32], a 10-item scale that assesses the extent of one’s resilience on a 7-point Likert scale ranging from 1 (strongly agree) to 7 (strongly disagree), with higher scores indicating greater resilience. The *α* coefficient for the current sample was 0.91.

#### Demoralization

Demoralization was measured using the *Demoralization Scale-II* (DS-II) [33], a 16-item scale that assesses demoralization on two dimensions, meaning and purpose and distress and coping ability, with higher scores indicating higher demoralization. Each item is rated on a 3-point Likert scale ranging from 0 (never) to 2 (often). Following Robinson et al. [33], the DS-II can be categorized in three ranges: low (scores 0 ~ 3), moderate (scores 4 ~ 10), and high demoralization (scores ≥ 11). The *α* coefficient for the current sample was 0.93.

### Statistical analyses

Statistical analyses were performed using SPSS version 27, setting the level of significance at 0.05.

First, the clinical characteristics of the participants, descriptive statistics, and bivariate correlations between the variables in the study were calculated.

Second, an analysis of variance (ANOVA) was performed to examine the nature of the association (e.g., linear or not) between demoralization and depressive symptoms using the DS-II as a continuous variable and the PHQ-9 as a categorical variable (i.e., low, moderate, and high depressive symptoms). In addition, a chi-square (*χ*^2^) test was calculated to identify potential differences between the percentage of patients with low, moderate, and high levels of depressive symptoms and demoralization and to compare the percentage of patients achieving cut-off scores for both clinical dimensions.

Subsequently, the associations between masculine self-esteem, depression, resilience, and demoralization were examined using a stepwise linear regression analysis, with demoralization as the outcome variable. In the linear regression, we entered demographics (age, education level, and relationship status) and disease severity dimensions (ISUP grade, Gleason score, and PSA) as confounders in step 1, masculine self-esteem and depression in step 2, resilience in step 3, and interaction terms in step 4. Masculine self-esteem and depression were entered in the model before resilience because they were assumed to be two dimensions potentially affecting demoralization, whereas resilience was assumed to be a moderating variable interacting with them and reducing their influence on demoralization. For this reason, two interaction terms were created (i.e., “Masculine Self-Esteem X Resilience” and “Depression X Resilience”). The independent variables (i.e., masculine self-esteem and depression) were centered, and each interaction was tested separately to avoid collinearity problems. We could not also control the regression models for ethnicity and sexual orientation because of the small number of non-Caucasian and non-heterosexual participants. Similarly, we could not include type of intervention because almost all participants underwent only radical prostatectomy and all other types of intervention had very low variance.

To assess the conditional effect of masculine self-esteem and depression on demoralization at different levels of resilience (− 1 standard deviation [SD], mean, + 1 SD), we used the PROCESS macro for SPSS, applying model 1 with 10,000 bias-corrected bootstrap samples [34].

Cohen’s *f*^*2*^ method was used as an indicator of effect size, according to which *f*^*2*^ ≥ 0.02, *f*^*2*^ ≥ 0.15, and *f*^*2*^ ≥ 0.35 represent small, medium, and large effect sizes, respectively. In addition, the variance inflation factor (VIF) was assessed to ensure that multicollinearity was not present. VIFs close to or above 5 can be considered acceptable values [35].

Finally, with G*Power, at a power of 95%, an *f*^*2*^ of 0.15, and an *α* of 0.05, at least 178 participants were needed. Thus, a sample of 197 participants was considered sufficient to perform stepwise linear regression analyses.

## Results

### Clinical characteristics of participants

The mean time since the last intervention for PCa was 1.58 years (*SD* = 1.47) and ranged from a few months to 8 years (from 2013 to 2021).

Most patients received surgery only (*n* = 188; 95.4%), while a smaller percentage of patients received surgery combined with ADT (*n* = 7; 3.6%), radiotherapy only (*n* = 1; 0.5%), and radiotherapy combined with surgery (*n* = 1; 0.5%).

Regarding the characteristic of histologically confirmed PCa, 22 (11.2%) patients had ISUP grade 1 and Gleason score 6, 33 (16.8%) had ISUP grade 2 and Gleason score 7 (3 + 4), 32 (16.2%) had ISUP grade 3 and Gleason score 7 (4 + 3), 86 (43.6%) had ISUP grade 4 and Gleason score 8, and 24 (12.2%) had ISUP grade 5, including 21 (10.7%) with Gleason score 9 and 3 (1.5%) with Gleason score 10. Finally, the median PSA level was 7.49 ng/ml (interquartile range [IQR] 5.15–11 ng/ml).

### Descriptive statistics and bivariate correlations

Means, standard deviations, ranges, and bivariate correlations between the variables analyzed (masculine self-esteem, depressive symptoms, resilience, and demoralization) are shown in Table 1. Pearson correlation results showed that all variables correlated with each other. Specifically, masculine self-esteem correlated moderately and positively with resilience, and strongly and negatively with depression and demoralization. Instead, depression correlated strongly and positively with demoralization.

**Table 1:** Descriptive statistics and bivariate correlations between masculine self-esteem, depressive symptoms, resilience, and demoralization

	1	2	3	4	Ranges	*M* ± *SD*
1. Masculine self-esteem	−				9 − 40	32.05 ± 7.25
2. Depressive symptoms	− 0.51***	−			0 − 24	5.41 ± 5.01
3. Resilience	0.34***	− 0.37***	−		1 − 7	5.64 ± 0.97
4. Demoralization	− 0.65***	0.67***	− 0.44***	−	0 − 31	6.55 ± 6.67

Note: *M* = mean; *SD* = standard deviation. ***p < 0.001

### Prevalence of depression and demoralization and percentage comparison

**Fig. 1:**
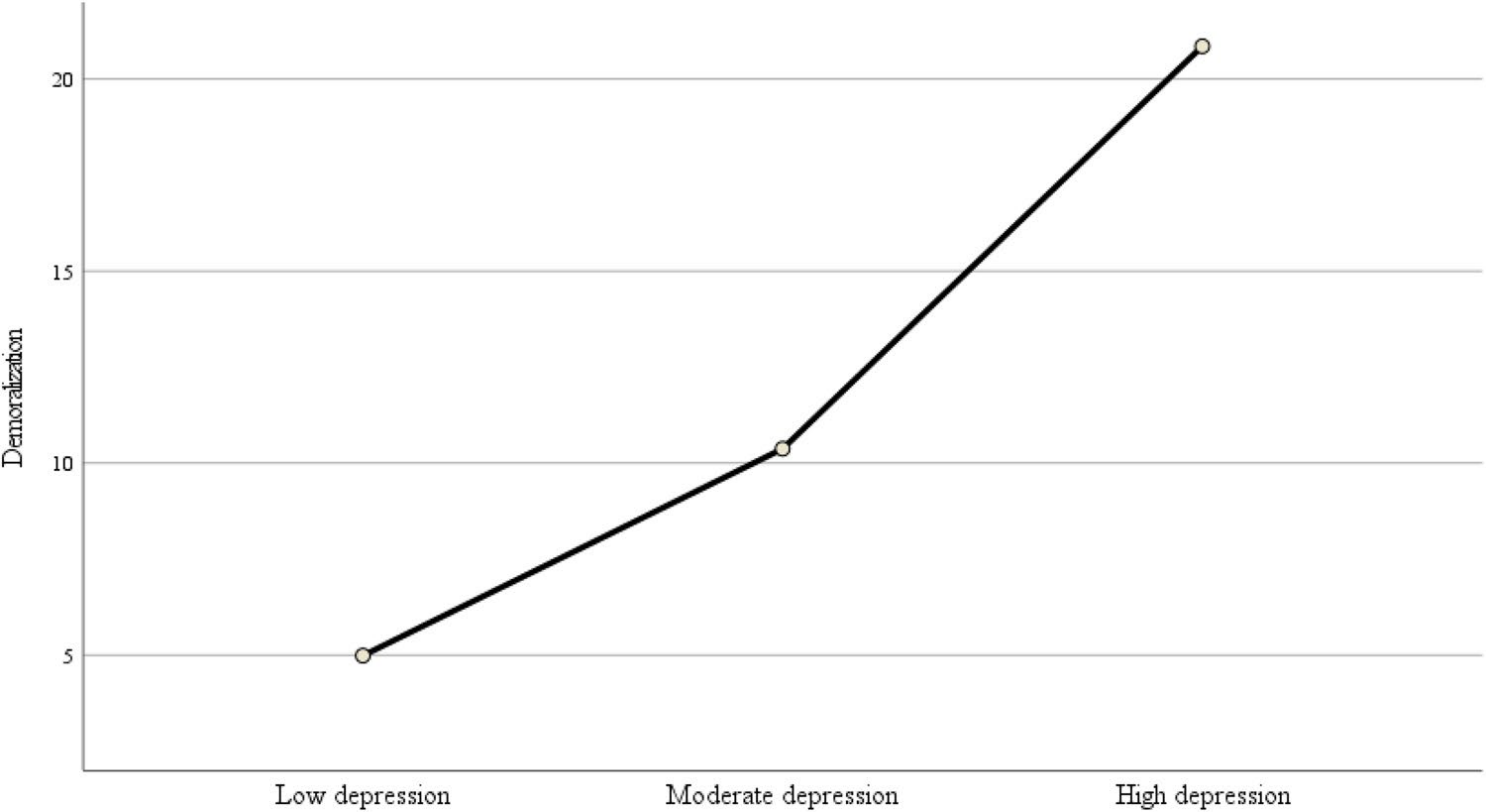
The association between demoralization and depression

As shown in Fig. 1 and in relation to Hypothesis 1, the ANOVA showed that depression scores increased linearly with the severity of demoralization (*F* = 20.92; *p* < 0.001). However, the percentage of patients with moderate or severe demoralization was significantly higher than the percentage of patients with moderate or severe depression (Table 2). Based on cut-off values reported by Kroenke et al. [29] for the PHQ-9 and by Robinson et al. [32] for the DS-II, 15.2% of the total sample had depressive symptoms, while 21.3% were demoralized.

**Table 2:** Prevalence of depression and demoralization and percentage comparison

	Ranges			Cut-off
	Low	Moderate	High	
Depression; *n* (%)	167 (84.8)	16 (8.1)	14 (7.1)	30 (15.2)
Demoralization; *n* (%)	85 (43.1)	70 (35.5)	42 (21.3)	42 (21.3)
	*χ*^*2*^ = 46.05***			*χ*^*2*^ = 43.38***

Note: Scores of ≥ 10 and ≥ 11 are cut-off points indicating high depression and demoralization, respectively [29, 33]. ***p < 0.001

### Associations between masculine self-esteem, depression, resilience, and demoralization

The results of the stepwise linear regression of demoralization on masculine self-esteem, depression, and resilience are shown in Table 3. All VIFs were acceptable, ranging from 1.01 to 1.76.

**Table 3:** Stepwise linear regression of demoralization on masculine self-esteem, depression, and resilience

	Model 1			Model 2			Model 3			Model 4		
	*b*	*B(SE)*	*95*% *CI*	*b*	*B(SE)*	*95*% *CI*	*b*	*B(SE)*	*95*% *CI*	*b*	*B(SE)*	*95*% *CI*
Age	0.03	0.03(0.07)	− 0.11,0.17	0.01	0.01(0.05)	− 0.08,0.10	0.01	0.01(0.05)	− 0.09,0.09	0.01	0.01(0.04)	− 0.08,0.09
Education level	− 0.16	− 2.56(1.13)	− 4.78,-0.33	− 0.07	− 1.02(0.75)	− 2.50,0.46	− 0.08	− 1.17(0.73)	− 2.61,0.26	− 0.08	-1.19(0.70)	− 2.58,0.20
Partner (no)	− 0.09	− 1.82(1.33)	− 4.46,0.81	0.01	0.16(0.89)	− 1.59,1.92	0.01	− 0.09(0.86)	− 1.80,1.62	− 0.01	− 0.09(0.84)	− 1.75,1.56
ISUP grade	− 0.32	− 1.72(1.76)	− 5.21,1.76	− 0.14	− 0.74(1.16)	− 3.04,1.55	− 0.14	− 0.76(1.13)	− 2.98,1.46	− 0.14	− 0.77(1.09)	− 2.91,1.39
PSA	− 0.11	− 0.07(0.05)	-0.16,0.02	− 0.06	− 0.04(0.03)	− 0.10,0.02	− 0.04	− 0.03(0.03)	− 0.09,0.03	-0.03	− 0.02(0.03)	− 0.08,0.04
Gleason score	0.33	2.26(2.25)	− 2.19,6.70	0.09	0.61(1.48)	− 2.32,3.54	0.11	0.75(1.44)	− 2.09,3.59	0.12	0.80(1.39)	− 1.94,3.55
Time of intervention	0.07	0.31(0.35)	-0.38,0.99	− 0.06	− 0.27(0.23)	− 0.72,0.20	− 0.04	− 0.18(0.23)	− 0.63,0.27	− 0.02	− 0.08(0.22)	− 0.52,0.35
MSE				− 0.47***	− 0.43(0.05)	− 0.54,-0.33	− 0.42***	− 0.38(0.05)	- − .49,-0.28	− 1.27***	− 1.16(0.22)	− 1.59,-0.73
PHQ				0.40***	0.53(0.08)	0.38,0.68	0.34***	0.46(0.08)	0.31,0.61	0.27***	0.37(0.08)	0.21,0.52
RS							− 0.19***	− 1.40(0.39)	− 2.18,-0.62	− 0.17**	− 1.24(0.38)	− 1.99,-0.48
MSE X RS										0.83***	0.14(0.04)	0.06,0.210
PHQ X RS										−	−	−
*R*^*2*^	0.05			0.59			0.62			0.65		
*F*	1.46			29.69***			29.69***			30.09***		
*F* for changes in *R*^*2*^	1.46			121.77***			12.63***			13.52***		
*f*^*2*^	−			1.47			1.64			1.84		

Note: *b* = standardized regression coefficient; *B* = unstandardized regression coefficient; *SE* = standard error; *CI* = confidence interval; *MSE* = masculine self-esteem; *RS* = resilience; *phq* = patient health questionnaire; *F* = *F* test; *R*^*2*^ = *R*-square. *f*^*2*^ = Cohen’s *f*^*2*^. ***p < 0.001; **p < 0.01; *p < 0.05

Neither demographics nor disease severity were associated with demoralization in the first step of the model. With regard to Hypothesis 2, the introduction of masculine self-esteem and depression in step 2 explained 59% of the variation in demoralization. Specifically, lower levels of masculine self-esteem and higher levels of depressive symptoms were associated with greater demoralization. The addition of resilience in step 3 explained an additional 3% of the variation in demoralization. Specifically, lower levels of resilience were associated with higher levels of demoralization. Finally, regarding Hypothesis 3, only the interaction term between masculine self-esteem and resilience was statistically significant and explained an additional 3% of the variation in demoralization. This suggests that resilience significantly moderated the association between masculine self-esteem and demoralization. In contrast, the interaction term between depression and resilience was not significant. The final statistical model for all dimensions explained 65% of the variance in demoralization, and with large effect sizes.

**Fig. 2:**
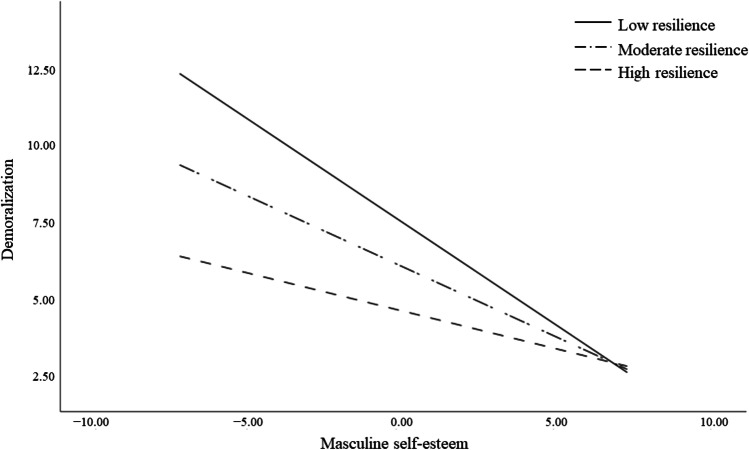
Interaction effect of masculine self-esteem by resilience on demoralization

Examination of the interaction plot showed that the effect of lower levels of masculine self-esteem on demoralization was significant for all resilience levels (low: *b* = – 0.67, *95% C.I.* [– 0.77, 0.57], *p* < 0.001; moderate: *b* = – 0.46, *95% C.I.* [– 0.55, – 0.36], *p* < 0.001; and high: *b* = 0.25, *95% C.I.* [– 0.37, – 0.12], *p* < 0.001) (Fig. 2), but stronger for those with low levels of resilience. In other words, resilience appears to be able to protect PCa patients from the effects that low masculine self-esteem may have on demoralization.

## Discussion

The current study examined the prevalence of demoralization in a group of PCa patients, distinguishing it from depressive symptoms. In addition, the associations of masculine self-esteem and depression with demoralization and the role of resilience as a buffer dimension protecting patients from the possible negative effects of masculine self-esteem and depression on demoralization were investigated. The results confirmed our hypotheses, with the exception of the interaction between depression and resilience on demoralization.

The group of results related to the first objective, i.e., the associations between depression and demoralization, seems to indicate that depression and demoralization are strongly and positively correlated, but that patients suffering from demoralization do not necessarily also have depressive symptoms. This is consistent with studies highlighting the differences between depression and demoralization as differentiated mental states [17, 22, 25]. Furthermore, in our sample, 15.2% of patients had significant depressive symptoms, which, although slightly lower, is still consistent with a previous study that reported the incidence rate of depression in PCa patients to be 17.07% [3]. Instead, we found that 21.3% of our sample had high demoralization. This percentage is slightly higher than that of Robinson et al. [17] in a sample of patients with different types of cancer (i.e., 13 ~ 18%), but very similar to that of Wu et al. [36].

Regarding the second hypothesis, we found that both masculine self-esteem and depressive symptoms were associated with demoralization, independent of demographics and disease severity. While the association between depressive symptoms and demoralization has been extensively studied in the cancer health literature and a robust association between these mental states has been demonstrated [17, 26, 37], the finding regarding the association between masculine self-esteem and demoralization is innovative, as masculine self-esteem has previously only been studied in relation to depression and quality of life in PCa patients [7, 10]. Previous studies reported that masculine self‐esteem is positively associated with optimistic capacity in men with PCa, whereas impaired masculine self‐esteem is negatively associated with emotional self‐reliance [38]. In addition, other studies have shown that personal masculine values can influence the way men respond to cancer, for example, by influencing help-seeking behaviors that might help them cope with emotional or sexual difficulties [8]. Indeed, traditional normative values of masculinity are associated with behaviors that increase health risk, such as avoidance, concealment, and emotional suppression [39]. Thus, the more the values and norms associated with masculinity are focused on the need to demonstrate “male power,” the more men with PCa will tend not to ask for help and thus be at higher risk for health problems. Therefore, it is plausible to hypothesize that men with PCa who experience problems related to their own masculinity are at higher risk of experiencing feelings of helplessness or meaninglessness, which are the core symptoms of demoralization. Regarding the loss of meaning, it is plausible to argue that the lack of meaning concerns the system of identity values associated with masculinity (e.g., being independent, self-reliant, strong, and sexually active) and around which the patient’s relational and emotional life as a man has been organized [40]. In relation to helplessness, it can also be argued that those who have more problems related to their masculinity may feel that the situation will not change over time and that their masculinity will not be what it once was. However, future studies should examine these interpretive hypotheses qualitatively, for example, by assessing the relationships between masculine self-esteem and demoralization through in-depth interviews.

Regarding the third hypothesis on the moderating role of resilience, we found that as resilience increased, the effect of low masculine self-esteem on demoralization decreased. This finding confirms a long tradition of cancer health studies emphasizing the fundamental role of resilience as a protective factor against the risk of developing adverse health outcomes [14–16]. Notably, our findings are consistent with those recently reported by Groarke et al. [41], who found that resilience moderates the relationship between stress and distress, and that the effects of masculine identity threat on adjustment diminish in the face of resilience [42]. However, in the current study, we only examined the individual characteristics of the construct, whereas there are several factors that contribute to the cancer patient’s resilience and thus mental health, including biological and social (e.g., social support) factors [43]. This may be one of the reasons why resilience did not moderate the association between depressive symptoms and demoralization in the current study. Indeed, previous studies have shown that social support is a critical factor in buffering depressive symptoms in cancer patients [44], but the RS does not measure this feature of resilience. Therefore, future studies should investigate resilience in PCa patients in more detail and analyze the role of different latent factors.

The results should be read in light of important limitations. First, the cross-sectional design of the study does not allow conclusive inferences about the directionality and causality of the relationships among the variables studied. Studies with a longitudinal design are needed to identify cause-effect relationships among depression, masculine self-esteem, resilience, and demoralization in PCa patients. Second, because of the composition of the sample, we were unable to assess the role of ethnicity, sexual orientation, and type of intervention. Future studies should strive to expand the sample and recruit patients with more diverse sociodemographic and clinical characteristics. In particular, because patients undergoing ADT are significantly more likely to report depressive symptoms than patients not undergoing the same treatment [3], future studies should compare the rate of demoralization in these patient groups and analyze possible differences. In addition, as this was a survivorship cohort of men who were nearly all treated by radical prostatectomy, the impact of the physical effects of such treatment (e.g., incontinence and erectile dysfunction) could be examined in a future study for their associations with demoralization. Third, the sample consisted only of Italian patients, and male norms and ideals may be subject to cultural biases. Therefore, it seems prudent to consider our results as potentially culturally biased.

Despite these limitations, our results may have some important clinical implications. The finding of a difference between depression and demoralization should prompt oncology clinicians and psychologists to assess both mental states in their PCa patients using questionnaires or clinical interviews. Although we have not examined the potential clinical consequences of demoralization, previous studies have shown that demoralization influences suicidal ideation more than depression [23, 24], and this makes the assessment of such a mental state necessary, as unrecognized demoralization may pose a serious health risk to patients. Oncology clinicians and psychologists should also explore the patient’s personal masculine values, as these may increase the risk of developing demoralization. For example, it is important to explore the fantasies and expectations that patients have built up regarding treatments they will have to undergo, as they are likely to associate the treatments and cancer in general with the loss of their masculinity, which in turn may increase feelings of demoralization. Finally, oncology clinicians and psychologists should also assess patients’ resilience and promote it when it proves inadequate [45].﻿ Indeed, previous studies have shown that overcoming cancer and its associated treatments can be an opportunity for personal growth and improved psychological well-being and mental health [46].

## Conclusions

This study highlights the crucial role of depression, masculine self-esteem, and resilience in their relations to demoralization in PCa patients. Our findings suggest that it is necessary to assess all these dimensions in the clinical setting to improve the mental health status of PCa patients.

## Data Availability

The data are available from the corresponding author on reasonable request.
